# Radiologist's liability for an erroneous report due to wrong labeling by juniors/para medics

**Published:** 2008-11

## Suggested precautions

1. Consulting radiologist must sign the report after ensuring that the procedure has been performed correctly.
2. Hospitals/nursing homes must develop and follow proper protocols in radiology and pathology departments to avoid wrong labeling.
3. During interventions, if the suspected tumor is not found at the suggested place, avoiding further exploration is advisable.

## Facts of the case

Patient developed suspected tumor on her back outside the spinal cord. Hospital (OP) conducted MRI scan. Scanned films were examined by the radiologist (OP) and the presence of tumor at D 10-11 position, outside the spinal cord was reported. On the basis of the MRI report, the patient was operated but no tumor was found at D 10-11. Another MRI, under the supervision of the same radiologist (OP), in the same hospital (OP), reported tumor at D 7-8 position. Hence, a second operation was performed and the tumor was removed.

## Patient's allegation/s of medical negligence

It was alleged that due to erroneous reporting of MRI scan, the patient had to undergo a second intervention.

## Doctor's defense

- In defense, the radiologist (OP) contended that there was no mistake on her part because there was a standard protocol followed in the hospital (OP) whereby the scan was actually performed by a technologist under the supervision of a Senior Resident Doctor, who is a qualified Radiologist. The radiologist (OP) was not required to routinely monitor the scan. Final films were documented from the computer monitor and she made report on the basis of the final labeled film put before her. It was because of the wrong labeling by the Senior Resident Doctor that the mistake occurred and therefore, she was not responsible.
- Hospital (OP) contended that the hospital was not liable for the deficiency in service rendered by the radiologist (OP) and the neurosurgeon (OP) as under its Rules and Regulations the entire responsibility of the treatment of the patient lay exclusively with the consultant under whom the patient was admitted.
- Neurosurgeon contended that he performed the operation on the basis of the MRI scan report to remove the tumor and hence he was not liable.

## Findings of the court

- The court observed that the radiologist (OP) was not vigilant in verifying whether the labeling made by the Senior Resident Doctor was correct and merely signed whatever the junior medical staff suggested and hence negligent.
- The court held that the hospital (OP) was liable for negligence as internal rules and regulations of the hospital (OP) were of no consequence to the patients who are admitted in the hospital. The court held that as the patient approaches the hospital for treatment and hence the primary liability in case of deficiency in service is that of the hospital. The court opined that if there was deficiency in service of any doctor, then, the hospital and erring doctor would be jointly and severally liable.
- The court did not pass any order on the alleged negligence of the neurosurgeon as he was dead before the appeal was decided.
- Hence the radiologist and the hospital (OP) were jointly held negligent.

